# Hyperthermia Induced by Near-Infrared Laser-Irradiated CsWO_3_ Nanoparticles Disintegrates Preformed Lysozyme Amyloid Fibrils

**DOI:** 10.3390/nano10030442

**Published:** 2020-02-29

**Authors:** Po-Sheng Hu, Natalia Tomasovicova, Hsiu-Jen Chou, Meng-Chang Li, Marek Vojtko, Katarina Zakutanska, Jozefina Majorosova, Shean-Jen Chen, Peter Kopcansky

**Affiliations:** 1 Institute of Photonic System, National Chiao Tung University, Tainan City 71150, Taiwan; 2 Institute of Experimental Physics, Slovak Academy of Sciences, Watsonova 47, 04001 Kosice, Slovakia; nhudak@saske.sk (N.T.); zakutanska@saske.sk (K.Z.); majorosova@saske.sk (J.M.); kopcan@saske.sk (P.K.); 3 Institute of Lighting and Energy Photonics, National Chiao Tung University, Tainan City 71150, Taiwan; jeffchou0510@gmail.com (H.-J.C.); as13572864@gmail.com (M.-C.L.); 4 Institute of Materials Research, Slovak Academy of Sciences, Watsonova 47, 04001 Kosice, Slovakia; mvojtko@saske.sk; 5 Institute of Imaging and Biomedical Photonics, National Chiao Tung University, Tainan City 71150, Taiwan; sheanjen@mail.ncku.edu.tw

**Keywords:** lysozyme amyloid fibrils, cesium tungsten oxide nanoparticles, self-assembled nanocomposite, hyperthermia, neurodegenerative diseases

## Abstract

This research study attempts to prove the concept of the applicability of hyperthermia to treating the lysozyme amyloid fibrils (LAFs)’s self-assembled fibrillary aggregates by a feedback-modulated temperature controller ranging from 26 °C to 80 °C, and separately, by near-infrared (NIR) laser-irradiated cesium tungstate (CsWO_3_) nanoparticle (NPs). The dependence of the final morphology of the amyloidal assembly on external heating and the photothermal effect of the NPs on treating the fibrillary assembly were investigated and analyzed. Experimentally, atomic force microscopy (AFM), optical stereoscopy, and scanning electron microscopy (SEM) were used primarily to ensure mutual interaction between LAFs and NPs, optically elucidate the surface contour and final fibrillary assembly upon the influence of thermal treatment, and further reveal fine-details of the optical samples. Finally, conclusive remarks are drawn that the fibrillary structures doped with the NPs exhibit an increasing degree of unique orthogonality. As the temperature rises, utter deformation of the dendritic structures of fibrillary assemblies at 70 °C was found, and NIR laser-irradiated CsWO_3_ NPs have been demonstrated to be useful in topically destructing pre-assembled LAFs, which may be conducive to the future development of neurodegenerative therapeutic techniques.

## 1. Introduction

The assembly of amyloid fibrils of many protein origins is a potential cause of many life-threatening diseases [1]. The amyloidal formation of fibrillary materials characterized by the morphology of reverse cross-double β-sheets composed of strands of polypeptides that are 4.8 angstroms apart is a unique structural signature of misfolded or partially misfolded protein aggregates. These amyloid fibrils, together with other aggregates—such as mutant tau protein and PTDP-17—have cytotoxic effects on cells and subsequently cause organ dysfunction, resulting in over 100 types of serious disorders [2], such as neuro-degenerative diseases [3], type II diabetes [4], and atherosclerosis [5]. Thus far, a handful of aggregates of mutant versions of proteins—including tau protein [6], lysozyme [7], insulin [8], and hungtingtin [9]—have been found to be pathologically complicated with the degeneration of cerebral tissue, the dysfunction of visceral organs, the requirement of increased insulin intake for diabetic patients, and the cytotoxicity induced by abnormal expansion of CAG codon of glutamine (an α amino acid of hungtingtin).

Fortunately, such aggregating phenomena of the amyloidal polypeptides, either toxic or non-toxic, can occur both in vivo or in vitro [10] and have driven a plethora of investigative efforts into methods that prohibit, disintegrate, or destruct such amyloidal formations [11]. Thus far, chemical and biological preventions have remained the most common methods to treating amyloidal fibrils-related pathologies, where ongoing efforts to discover drugs that retain the stability of soluble proteins, molecularly annihilate and prevent the formation of fibrils and Aβ proteins, and inhibit biological catalysis of Aβ peptides are the main strategic schemes in helping prolong the health of organs [11,12]. More recently, nanomaterial system-based therapeutic agents, coated or uncoated, with the advantages like large surface-to-volume ratio, functional versatility, and biological compatibility have drawn considerable attention for treating fibrillary aggregate–induced complication [13,14]. For instances, bared nanomaterials such as graphene nanosheets, zinc oxide nanoparticles (NPs), or Fe_3_O_4_ NPs have already been demonstrated [15,16]. Additionally, anti-Aβ monoclonal antibody-coated iron oxide NPs and glutathione-wrapped and curcumin-wrapped gold (Au) NPs have proven to be capable of inhibiting fibrillary formation [17,18,19]. To further reveal the fundamental mechanisms governing such destructive effects, Liao et. al. and Sudhakar et. al. found mutual electrical attraction to be the main driving forces when the surface of the NPs, whether coated or non-coated, are negatively charged [20,21]. Besides electronic forces, an alternative method of microwave hyperthermia induced by exposing AuNPs to AC magnetic field for eight hours has been verified, with its toxic effects on the aggregates of Aβ_1-42_, reducing the fibrils into shortened and agglomerated debris [22]. Although most of the aforementioned studies presented quantification of the NP-treated suspended fibrils with the incorporation of a thioflavin T fluorescence assay, which presents fluorescence level of the aggregating fibrils, the ability of the fluorescent dye to destroy unique features of metastable protein mutants has been verified, thus lowering the toxic effects of amyloid β aggregates [23]. Moreover, despite the acquisition of electron microscopy images of lysozyme amyloid fibrils (LAFs) before and after the microwave irradiation was carried out, the dynamic process of such fibrillary disintegration due to the imposition of thermal energy is still lacking [24]. 

Hence, the research presented herein is intended to investigate, morphologically, the physical condition of hyperthermia induced by external heating and NIR-irradiated CsWO_3_ NPs, required for disintegrating amyloid fibrils originated from hen egg white lysozyme (HEWL) by ruling out any use of fluorescence assay. To shed light onto any potential interplay between the LAFs and NPs, AFM images were acquired to determine the degree of binding affinity and ensure the presence of the NPs in the fibrillary nanocomposite, which is vitally important for photothermal destruction of the fibrillary amyloids. Lysozyme protein is in a globular monomeric form composed of 129 amino acids; has high degree of similarity in tertiary structure, protein folding characteristics, and functional catalysis of peptidoglycans to its human counterparts; can form protofibrils that cooperatively assemble into a large macromolecular complexes through oligomer fusion; and is easily soluble in water, rendering it a suitable in vitro model for biomedical study [25,26]. After preparation of LAFs solution and depositing it on glass substrate, the thermal treatment is applied to the sample that undergoes the drying process to elucidate the conformational effects of copolymer-coated CsWO_3_ NPs on the thermally treated LAFs, and to assess the suitability of CsWO_3_ NPs as the photothermally triggering agents for amyloidal disintegration. Experimentally, composite samples were prepared for AFM to interrogate whether these two types of material species, biological and inorganic, could interact and to elucidate the mutual interaction at molecular level. Additionally, an array of temperature ranging from 26 °C to 80 °C is applied to treating the LAFs doped with and without the CsWO_3_ NPs (while being optically recorded). The samples were subsequently examined by SEM. Finally, the application of NIR irradiation upon NP-doped LAFs was carried out to evaluate the ability of the NPs to disintegrating LAFs. 

## 2. Material and Methods 

### 2.1. CsWO_3_ Nanoparticle Suspension

**Figure 1:**
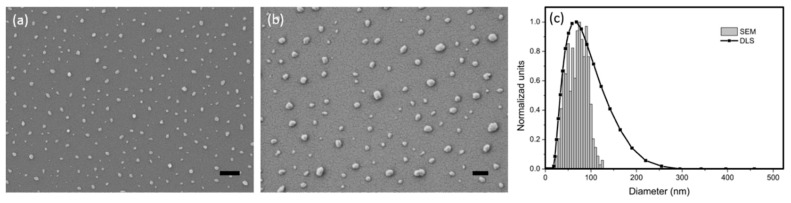
Characterization of CsWO_3_ nanoparticle (NPs). (**a**,**b**) Scanning electron microscopy (SEM) images and (**c**) distribution profile of feature sizes of the NPs are illustrated. The scale bars shown on (**a**) and (**b**) are 500 nm and 200 nm, respectively.

CsWO_3_ NPs are a type of nanocrystal that strongly absorb electromagnetic waves ranging from 800 nm to 2400 nm; convert the NIR radiation into heat, which is highly dependent on its crystalline structure and particle sizes; and employed in this research to exploit its photothermal properties for treating LAFs. The suspension of commercial copolymer-covered CsWO_3_ NPs, which is coated by a molecular fluid consisted of copolymer with pigment affinic group to prevent NP aggregation in deionized (DI) water, was obtained from Justnano Corporation (Tainan, Taiwan) and has a concentration of 108 mg/ml. Figure 1 depicts (a, b) surface morphology in the SEM image and (c) the profile of size distribution determined by a zetasizer, indicating well-distributed and suspended particles in the solution bath with an average particle size of 58.2 nm. By doping the NPs to the LAFs through a mixing process alongside illumination with an NIR laser beam operating at the wavelength of 980 nm, the feasibility of fibrillary disintegration by NIR-irradiated NPs can be assessed.

### 2.2. Sample Preparations

In this research, samples for AFM and SEM were prepared to reveal the interaction between the NPs and LAFs and to examine the dynamic morphogenesis and final structures of the LAFs and fibrillary nanocomposites undergone thermal treatment. The procedure of sample preparation began with the culturing of lysozyme amyloid fibrils derived from hen egg white (HEWL) (lyophilized powder, lot number Roche-10837059001, >23,000 units per mg protein, Sigma Aldrich, St. Louis, MO, US). Other chemicals like glycine (50046, Sigma Aldrich, St. Louis, MO, US) and HCl (9535-01, J.T. Baker Chemicals, Pittsburgh, PA, US) are all of analytic grades. By dissolving proper amount of HEWL powder in the mixture of 1 ml of 0.2 M glycine-HCl with pH of 2.2 and 4.7 mg of 80 mM NaCl (s9888, Sigma Aldrich, St. Louis, MO, US), the HEWL solution with final concentration of 5 mg/ml was produced, and subsequently boiled at 65 °C for 2 h in an enclosed flask under magnetic stirring with the constant speed of 250 round per minutes (rpm). To proceed with optical interrogation and SEM imaging, 98.5 μL of pure fibrils solution and 1.5 μL of NP solution were mixed well in a 5 ml beaker by micro-centrifuge, and 10 μL of the mixture was dropped onto a glass slide and treated with a negative feedback-controlled heater device at the set temperature before being sent to image acquisition. Regarding AFM imaging, 10 μL of the mixture was dropped onto a mica slide and washed drop-by-drop with DI water to retain a thin film-like amount of sample on the substrate.

### 2.3. UV-VIS-NIR Optical Absorbance Spectroscopy

Optical absorbance spectra of the NPs with an array of concentration, 1.5 mg/ml, 1 mg/ml, and 0.5 mg/ml spanning the wavelength range from 300 nm to 1300 nm were measured by ultraviolet-visible-near-infrared (UV-VIS-NIR) optical absorbance spectroscopy (V-750, Jasco, Tokyo, Japan). To acquire the spectra, 2.5 ml of NP solution was prepared by diluting NP solution with proper amount of DI water, filled in plastic cuvettes, and placed in the sample holder of the spectroscopy.

### 2.4. Optical Stereoscope

An epi-illumination-based optical stereoscope (SMZ745T, Nikon, Tokyo, Japan) equipped with a camera is used to acquire large-area images of entire drops of pure NPs and fibrils as well as their mixture. Stereoscopic images allow the visualization of any protrusion of objects such as fibrillary formation as well as relevant changes on the superficial surface of the structures. 

### 2.5. Atomic Force Microscopy

To reveal whether there is indeed a mutual interaction between LAFs and CsWO_3_ NPs, AFM imaging was employed to visualize the tomographical distribution of the mixture nanocomposites. After the preparation of AFM sample as explained in the above sub-section, it is properly placed in the aerated chamber platform of AFM (Veeco di Innova, Bruker AXS Inc., Madison, WI, USA). The tapping-mode of the AFM and the antimony-doped silicon tips (Bruker, Billerica, MA, USA) covered with a thin layer of aluminum for maximal reception of laser beam signal were used for image acquisition in an ambient environment with humidity of 30–40% and temperature of 26 °C. Analysis of feature dimension of LAFs and nanocomposites was carried out by Gwiddion analytic software.

### 2.6. Scanning Electron Microscopy

After optical interrogation, the final surface morphology of the thermally treated fibrillary assembly doped with/without the NPs was examined using ultra-high resolution field-emission SEM (SU-5000, Hitachi, Tokyo, Japan). The same samples from the optical experiment were mounted onto the chamber ceiling of SEM, deposited with a layer of gold about a few tens of nanometer thin for the enhancement of scattered electron signal, and data acquisition then proceeded.

### 2.7. Zeta Potential Analyzer

Zeta potential analyzer, the zetasizer from Malvern Co. (nanoZS, Cambridge, UK) was employed to measure the zeta potential for the solution of pure fibrils, NPs and their mixture nanocomposite, which was done at 25 °C. 

## 3. Results

A handful of previous research have reported the phenomena of thermal denaturation of aggregating protein complexes [22,27]. This research aimed to optically elucidate the in vitro morphogenesis of the lysozyme fibrillary assembly incorporated with CsWO_3_ NPs, investigate the effect of external heating through direct increase of environmental temperature or the NIR laser-irradiated NPs on the LAFs and nanocomposites, and assess the potential of the NPs on the treatment of the pre-assembled amyloidal fibrils. 

**Figure 2:**
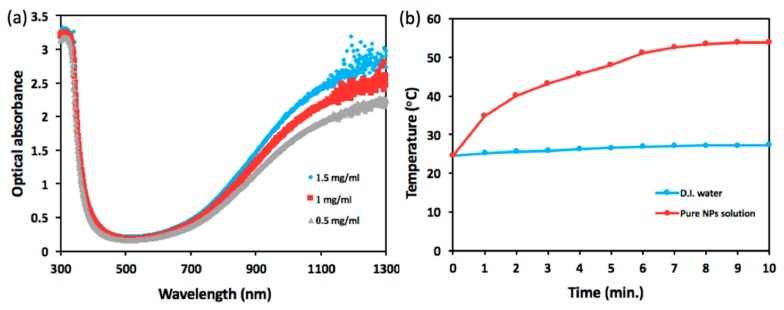
Optical measurement. (**a**) Optical absorbance spectra and (**b**) temporally dependent temperature of CsWO_3_ NPs solution illuminated by near-infrared (NIR) laser beam with average power density of 6.25 W/cm^2^ are illustrated.

Figure 2 presents optical characterization of the NPs including photo-absorbance and ability of photothermal conversion. As shown in Figure 2a, cesium tungsten oxide NP, a type of hexagonal tungsten bronze crystalline compound, is highly absorptive to optical wavelength spanning from 800 nm to 1300 nm, exhibiting a sharp contrast to the range of visible wavelength from 400 nm to 700 nm and strongly dependent on NP concentration. In Figure 2b, the temperature of NIR laser-illuminated NP solution tops 53.9 °C from 24.6 °C, whereas NIR illumination upon DI water engenders a rise in temperature from 24.6 °C to 27.3 °C over the course of 10 min.

**Figure 3:**
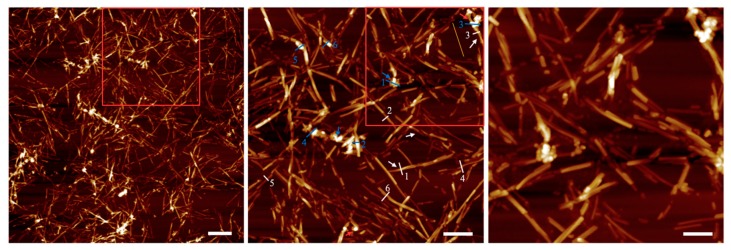
AFM images of CsWO_3_ NPs-doped LAFs with increasing magnification indicated by scale bars of 500 nm, 250 nm and 125 nm from left to right, are illustrated. Blue and white arrows mark the corresponding location of NP clusters and fibrils, and the dimension analysis of LAFs and nanocomposites are pointed out by enumerated white and blue lines, respectively.

**Figure 4:**
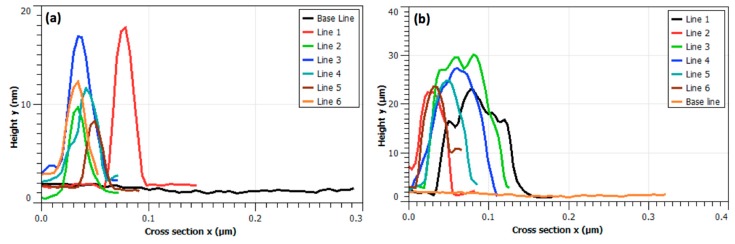
Dimensional analysis. Spatial profiles of height y across the surface morphologies of (**a**) LAFs and (**b**) nanocomposites indicated by the enumerated white and blue lines in Figure 3, where yellow line is the base line for both analysis, are presented.

To unravel the outcome of mutual interplay between NPs and LAFs, AFM images were acquired upon the dried mixture sample deposited on a cleaved mica sheet, and shown in Figure 3, in which fibrillary affinity of the NPs is confirmed by the sharp contrast of agglomeration of NPs. Also, the numerical analysis on the feature thickness of LAFs and nanocomposites is presented in Figure 4 where, the NP agglomeration, in general, exhibits larger sizes than the LAFs. 

**Figure 5:**
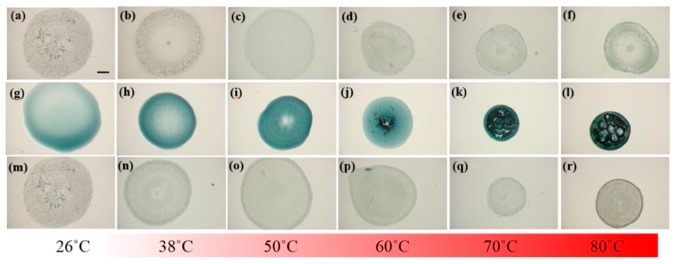
Large-area stereoscopic images of thermally treated (**a**–**f**) LAFs, (**g**–**l**) CsWO_3_ NPs, and (**m**–**r**) their mixed nanocomposites are illustrated. The scale bar shown in (**a**) is 1 mm, applicable to all images in this figure.

In addition to the characterization of NPs and verification of affinity between NPs and LAFs, thermal treatment upon the pure LAFs, NPs and their mixture composites at the designated temperatures 26 °C, 38 °C, 50 °C, 60 °C, 70 °C, and 80 °C were examined using a LED-based stereoscopic microscopy, where images of the top views of the samples were acquired and shown in Figure 5 to ensure that the morphogenesis of the fibrillary nanocomposite is rendered by the fibrillary interaction with NPs rather than the results of heating effects upon the pure ingredients. The intrinsic color of CsWO_3_ NPs is dark blue, which explains the color of its solution on the second row where the distribution of the NPs is uniformly across the entire droplet without any noticeable aggregation. With 1.5 μL of the NP solution added to 98.5 μL of LAFs solution, the nanocomposites contain merely 1.5% of NPs in the mixture, which is the reason for the bluish particles being invisible to the naked eye. As can be seen from the top row of the figure, regardless of the temperature, the influence of thermal treatment does not cause any significant physical distortion on the entire sample droplets, whereas, in the middle row, the increase in treatment temperature not only shrank the coverage of the NP solution on the substrates but also generated an air-bubble-like void once the temperature reached 60 °C. Evidently, in the bottom row of nanocomposites, the contour of the droplets treated with all temperature are not altered. Overall, except the untreated LAFs and nanocomposites assembled in the ambient aerated condition, where the even distribution of the assembled structures over the entire droplets is pronounced, the structures of the other samples tend to conform to the edges of the droplets. 

**Figure 6:**
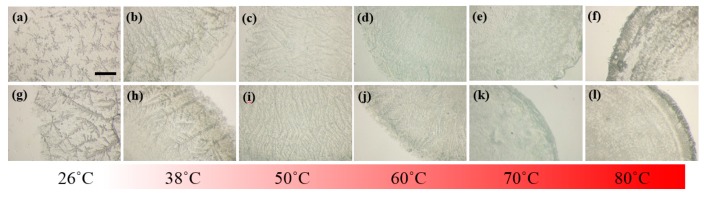
Stereoscopic images of the edges of (**a**–**f**) pure and (**g**–**l**) CsWO_3_ NP-doped LAFs treated at various temperature. The scale bar shown in (**a**) is 1 mm, applicable to all images in this figure.

To shed light onto the detailed morphology of the LAFs doped with or without the NPs, zoomed-in images on the edges of the samples were acquired and shown in Figure 6. With a glimpse into the assemblies of LAFs and nanocompsites dried at ambient temperature, it can found that the dendritic structures of pure LAFs is denser and smaller than those of the nanocomposites. As the temperature of treatment raises to 38 °C, both the self-assembled LAFs and nanocomposites exhibit slightly larger dendritic structures, and interestingly, contrary to the randomly disposed dendrites of the LAFs, the arrangement of the main branches of dendrites of the nanocomposites tends to orient perpendicularly to the rim of the droplet. At 50 °C, the morphology of both types of samples become smaller and thinner structures, and one major difference between the two is nanocomposites’ high degree of vertical organization, a sharp contrast compared to the LAFs’ randomness in fibrillary orientation, which agrees well with the previous results that the morphological pattern, structural orientation and size of LAFs undergo drastic transformation at 51.2 °C [24].

**Figure 7:**
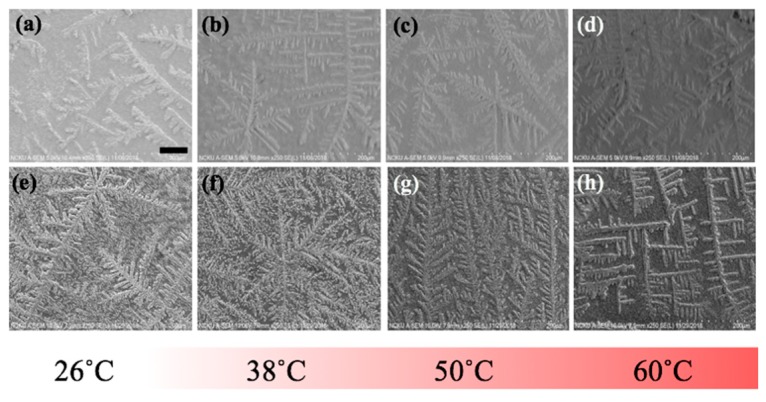
SEM images acquired at the edges of (**a**–**d**) pure and (**e**–**h**) CsWO_3_ NP-doped LAFs treated with an array of designated temperature; the scale bar shown in (**a**) is 80 μm, applicable to all images in this figure.

**Figure 8:**
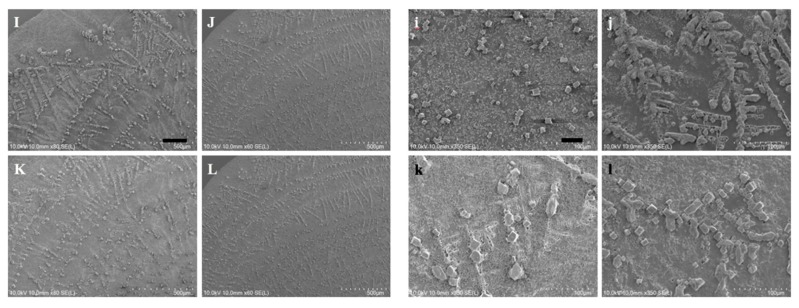
SEM images of (**I**,**J**) LAFs and (**K**,**L**) fibrillary nanocomposites, treated with (**I**,**K**) 70 °C and (**J**,**L**) 80 °C, as well as (**i**, **j**, **k**, **l**) the corresponding zoomed-in scope of views are illustrated on left-hand and right-hand panels with scale bars of 200 μm and 40 μm, respectively.

**Figure 9:**
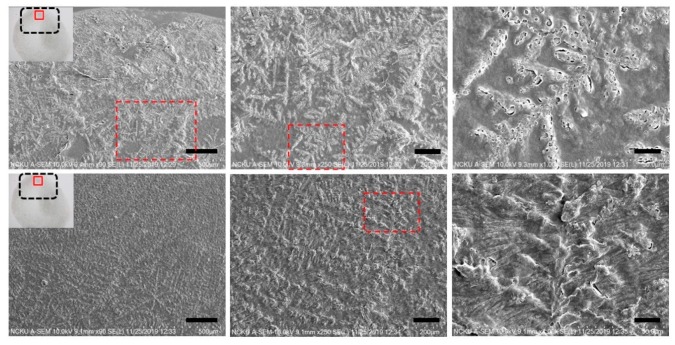
SEM images of the final constructs of LAFs and fibrillary nanocomposites after treatment of NIR laser-irradiation, are shown in the top and bottom rows, correspondingly. Scale bars from left- to right-hand panels are 200 μm, 60 μm and 15 μm, respectively; optical power density used in the sample treatment is 1.56 W/cm^2^.

To further elucidate the influence of thermal treatment upon the fibrillary assemblies, SEM images of the same samples from the optical experiment were acquired and presented in Figure 7 where the characteristic features of the LAFs and nanocomposites such as the sizes, transitory morphology and morphological arrangement as a function of temperature from 26 °C to 60 °C can be visually confirmed. Likewise, to verify that 70 °C and above may be the pivotal temperature in destructing the fibrillary assemblies, resultant morphologies for the samples of 70 °C and 80 °C are presented separately in Figure 8, where some leftover debris are exhibited. To assess NIR-irradiated NPs’ ability to destroy the LAFs, the LAFs and the nanocomposites, of which the nominal drying duration for both is about 10 min, were irradiated by a NIR laser beam with central wavelength of 980 nm and beam dimension of 4 mm by 4 mm for 15 min. Images were acquired with SEM imaging and are presented in Figure 9, where scale of resolution reduces from left to right, and each zoomed-in image can be traced from the red dotted rectangle of preceding magnification.

## 4. Discussion

Since the early nineteenth century, when sources of electromagnetic waves were first discovered [28], medical hyperthermia has been useful in the treatment of a handful of diseases such as cancer, inflammatory disorders, Peyronie’s diseases, and fibromyalgia syndrome [29,30,31]. From somatic point of view, thermal treatment allows the healing of wound and relief of pain and stress through a number of means including, but not limited to, accelerated bio-metabolism and an increase in the velocities of nerve conduction and blood flow [30]. Molecularly, moderate thermal treatment up to 40 °C can facilitate proliferation of some cell species such as blood mononuclear cells and lymphocytes, consequentially increasing the secretion of immune responsive proteins interfereon and thymidine that combat infection and inflammation [32,33]. Additionally, anti-inflammatory effects of hyperthermia through alteration of the sequence of pro-inflammatory genes, reduction of immune proteins, and prevention of biochemical process such as phosphorylation have also been reported [34,35,36]. 

Contrary to the beneficial effects in the aforementioned medical practice the thermal exposure upon brain has been proven deleterious and, in some cases, harmful to the functions of cerebral molecular expression [37,38]. Regarding the complication of neuronal degeneration like Alzheimer’s and Huntington diseases caused by the toxic aggregation of amyloid β peptides alongside other aggregating molecules of genetically abnormal tau protein and FTDP-17, previous research indicated that treating neuron cells with mild hyperthermia up to the range of 42 °C to 45 °C reduces amyloidal protein-induced toxicity via the induction of heat shocked protein and down-regulation of amyloid proteins in the phosphorylated states [39,40]. Also, Bastus et. al. demonstrated the successful destruction of toxic human amyloid peptide sequence Aβ_1-42_ by incubating the protein peptides with gold nanoparticles and then remotely heating the particles by microwave irradiation [22]. From the perspective of assembled LAFs, although one study reported the commencing denaturation of native state of fibrils at temperature above 51 °C by monitoring the transient Fourier-transform infrared spectral intensities, the morphological process of such denaturation and condition of utter destruction has yet to be illustrated [24]. 

In the present research, experimental protocols were designed to investigate the transitory process of thermal treatment upon disintegration of LAFs resembling the deposits of partially misfolded protein in vitro in order to elucidate morphologically the exposure of the LAFs and nanocomposites doped with CsWO_3_ NPs to an array of designated temperature and to assess whether the photothermal property of the NPs is capable of disintegrating the assembled fibrils.

This study is commenced with optical and morphological characterization of the NPs to verify the distribution of its feature sizes, and capabilities of NIR absorption and photothermal conversion, which are readily verified in result section. AFM imaging of the interaction between LAFs and NPs is illustrated in Figure 3 to ensure their mutual attraction. The enhancement of sharp contrast by the attachment of copolymer-coated NPs to the surface of LAFs, which occurs during the drying process and is indicated by blue arrows, is pronounced. The association of the NPs to the LAFs is a random event, and the slender appearance of the LAFs indicated by white arrow is also exhibited. In Figure 3a, a scope of view of 5 μm by 5 μm overlooking the nanocomposite clearly presents the aggregates of the NPs onto some LAFs, and an almost null unattached NPs can be observed. By zooming into the red rectangle-demarcated region shown in 3(a), the pavement of NPs on the surface along the length of fibrils is clear, and the size of the nanocomposites varies from one fibril to another depending on the degree of NP aggregation.

Further magnification of the red rectangle in 3(b) is presented in 3(c), which further illuminates the aggregates of NPs with uniformly layered concentric structures on segments of fibrillary surfaces as the thickened smooth outfit of NPs wraps around the fibrils are apparent, and large segments of the fibrils are covered with the clusters of NPs, which is drastically different from our previous result with Fe_3_O_4_ NPs [41].

Additionally, the analysis of feature sizes of LAFs and nanocomposites was carried out by illustrating the spatially dependent height profiles along the lines marked across the main feature dimension of interest, and is depicted in Figure 4, which is tabulated in Table 1, suggesting that an affinity between the material species is certain, incorporation of the NPs with the LAFs does render a drastically distinct material species, and any subsequent action, photo-irradiation in our case, may cause consequential effects on the fibrillary assembly.

To explain such adhering interaction between LAFs and NPs, our measurement of zeta potential for the LAFs, NPs and fibrillary nanocomposite indicate the respective values of 44.8 mV, –26.2 mV, and 21 mV, and the opposite polarity of zeta potentials of NPs and fibrils implies mutually attractive force the main driven mechanism of the interaction between the two material species. 

With the same sets of samples from the optical experiment, SEM images in Figure 7 were acquired to unravel additional details into the edge region of the samples, where the dendritic structures are formed. As can be observed from the figure, particularly in the case of the nanocomposite, the general trend of gradual transformation from randomly organized dendritic structure at 26 °C to high degree of orthogonality at 60 °C is elucidated, whereas, such transformative orthogonality is not observed in the case of pure LAFs. By cranking up the external temperature, an utter destruction of the LAFs and nanocomposites alongside the remains of crystalline structure of salt was observed, respectively, in Figure 8i,k when the treatment temperature reaches 70 °C, and surprisingly, as can be seen from Figure 8j,l, the remains of salt-based dendritic morphology of both types of samples found at 80 °C are structurally more substantial. The purpose of adding salt in the fibrillary model is to improve LAF’s stability up to at least one year of shelf-life time as previously investigated in our past study where, regardless of the presence of salt, the aggregating action of lysozyme protein and formation of LAFs are not affected [42]. Thus, the resultant LAFs morphologies by the treatment of external thermal influence does not alter significantly even in the presence of salt. 

Having elucidated the thermally induced morphological changes, the question of whether photothermal effects of CsWO_3_ NPs is effective in destructing the fibril aggregation was interrogated by irradiating the LAFs and nanocomposites during the entire duration of drying process with the ambient environment conditioned at temperature of 26 °C and humidity ranging between 40% to 50%. The SEM images shown in Figure 9 depict the resultant morphology of LAFs and the nanocomposites after NIR irradiation, and the image of highest magnification reveals that the intrinsic dendritic structure of the irradiated LAFs is conspicuous and remains intact against the photothermal influence, whereas, as evidently shown in the figure of preceding magnification, the dendritic structure of the irradiated nanocomposite appears to be topically tarnished and distorted. Such fibrillary destruction, as it was demonstrated in our past studies [15,41] where disintegrating LAFs by size-dependent and electrostatic stabilization effects of Fe_3_O_4_ NPs had been achieved, does not depend on the uniformity of particle distribution, suggesting effectiveness of NIR laser-irradiated CsWO_3_ NPs in treating LAFs, which may further be applied to other types of amyloid fibrils of pathological origins.

In summary, several remarks are drawn. First, thermal treatment, even at slightly above human body temperature (38 °C), is effective in tuning the morphology of the LAFs and nanocomposites, and the increasing degree of unique orthogonality of the nanocomposites as the treatment temperature increases from 26 °C to 60 °C was revealed, which was not found in its pure LAF counterpart and may be used for designing bio-inorganic devices with optical and electrical manipulation; second, complete destruction for both types of samples at 70 °C alongside the crystalline remains of salt was verified; third, destruction of the NP-incorporated LAF when irradiated with NIR laser was demonstrated, potentially viable for future experimental trials in treating neurodegenerative diseases. 

## 5. Conclusions

This research investigated, morphologically, the temperature-dependent morphogenesis of the self-assembled LAFs and nanocomposites doped with CsWO_3_ NPs and assessed the applicability of photothermal property of the NPs to treating the aggregates of LAFs. Expressly, unique orthogonality of the formation of thermally treated fibrillary nanocomposites occurs at 60 °C, the morphologies of LAFs and composites are completely annihilated and left with crystalline remains of salt, and the NIR-irradiated CsWO_3_ NPs successfully destruct the preformed LAFs. Finally, a conclusion is drawn that the orthogonality of thermally treated nanocomposites and irradiation of NPs may be conducive to the development of advanced optical devices and therapeutic techniques against fibrillary aggregation-induced amyloidal diseases. 

## Figures and Tables

**Table 1:** Estimation of cross-sectional feature sizes of LAFs and nanocomposites.

No. of Line	LAFs (nm)	Nanocomposites (nm)
1	38.7	126.8
2	33.9	50.7
3	46.6	107.2
4	45.2	106.3
5	31.8	83.5
6	41.8	54.8
Average	39.6	88.2

## References

[1] Rambaran R.N., Serpell L.C. (2008). Amyloid fibrils: abnormal protein assembly. Prion.

[2] Kelly J.W. (2002). Towards an understanding of amyloidogenesis. Nat. Struct. Biol..

[3] Comellas G., Lemkau L.R., Nieuwkoop A.J., Kloepper K.D., Ladror D.T., Ebisu R., Woods W.S., Lipton A.S., George J.M., Rienstra C.M. (2011). Structured regions of a-synuclein fibrils include the early-onset Parkinson’s disease mutation sites. J. Mol. Biol..

[4] Westermark P., Andersson A., Westermark G.T. (2011). Islet Amyloid polypeptide, islet amyloid, and diabetes mellitus. Physiol. Rev..

[5] Mucchiano G.I., Jonasson L., Haggqvist B., Einarsson E., Westermark P. (2001). Apolipoprotein A-I-Derived amyloid in atherosclerosis. Am. J. Clin. Pathol..

[6] Selkoe D.J. (1991). The molecular pathology of alzheimer’s disease. Neuron.

[7] Pepys M.B., Hawkins P.N., Booth D.R., Vigushin D.M., Tennent G.A., Soutar A.K., Totty N., Nguyen O., Blake C.C.F., Terry C.J. (1993). Human lysozyme gene mutations cause hereditary systemic amyloidosis. Nature.

[8] Stefani M., Dobson C.M. (2003). Protein aggregation and aggregate toxicity: new insights into protein folding, misfolding diseases and biological evolution. J. Mol. Med..

[9] Zheng Z., Diamond M.I. (2012). Huntington disease and the huntingtin protein. Prog. Mol. Biol. Transl. Sci..

[10] Fandrich M. (2007). On the structural definition of amyloid fibrils and other polypeptide aggregates, *Cell Mol*. Life Sci..

[11] Eisenberg D., Jucker M. (2012). The Amyloid state of proteins in human diseases. Cell.

[12] Schilling S., Zeitschel U., Hoffmann T., Heiser U., Francke M., Kehlen A., Holzer M., Hutter-Paier B., Prokesch M., Winisch M. (2008). Glutaminyl cyclase inhibition attenuates pyroglutamate AB and Alzheimer’s disease-like pathology. Nat. Med..

[13] Estevez A.Y., Erlichman J.S. (2011). Cerium oxide nanoparticles for the treatment of neurological oxidative stress diseases. ACS Symposium Series.

[14] Carroll R.T., Bhatia D., Geldenhuys W., Bhatia R., Miladore N., Bishayee A., Sutariya A. (2010). Brain-targeted delivery of tempol-loaded nanoparticles for neurological disorders. J. Drug Target.

[15] Bellova A., Bystrenova E., Koneracka M., Kopcansky P., Valle F., Tomasovicova N., Timko M., Bagelova J., Biscarini F., Gazova Z. (2010). Effect of Fe_3_O_4_ magnetic nanoparticles on lysozyme amyloid aggregation. Nanotechnology.

[16] Ban D.K., Paul S. (2016). Nano zinc oxide inhibits fibrillary growth and suppresses cellular toxicity of lysozyme amyloid. ACS Appl. Mater. Interfaces.

[17] Antosova A., Gazova Z., Fedunova D., Valusova E., Bystrenova E., Valle F., Daxnerova Z., Biscarini F., Antalik M. (2012). Anti-amyloidogenic activity of glutathione-covered gold nanoparticles. Mater. Sci. Eng. C.

[18] Skaat H., Chen R., Grinberg I., Margel S. (2012). Engineered polymer nanoparticles containing hydrophobic dipeptide for inhibition of amyloid-b fibrillation. Biomacromolecules.

[19] Palmal S., Maity A.R., Singh B.K., Basu S., Jana N.R. (2014). Inhibition of amyloid fibril growth and dissolution of amyloid fibrils by curcumin-gold nanoparticles. Chemistry.

[20] Liao Y.H., Chang Y.J., Yoshiike Y., Chang Y.C., Chen Y.R. (2012). Negatively charged gold nanoparticles inhibit alzheimer’s amyloid-b fibrillation, induce fibril dissociation, and mitigate neurotoxicity. Small.

[21] Sudhakar S., Kalipillai P., Santhosh P.B., Mani E. (2017). Role of surface charge of inhibitors on amyloid beta fibrillation. J. Phys. Chem. C.

[22] Bastus N.G., Kogan M.J., Amigo R., Grillo-Bosch D., Araya E., Turiel A., Labarta A., Giralt E., Puntes V.F. (2007). Gold nanoparticles for selective and remote heating of B-amyloid protein aggregates. Mater. Sci. Eng. C.

[23] Alavez S., Vantipalli M.C., Zucker D.J.S., Klang I.M., Lithgow G.J. (2011). Amyloid-binding compounds maintain protein homeostasis during ageing and extend lifespan. Nature.

[24] Meersman F., Heremans K. (2003). Temperature-induced dissociation of protein aggregates: accessing the denatured state. Biochemistry.

[25] Swaminathan R., Ravi R.V.K., Kumar S., Kumar M.V.S., Chandra N. (2011). Lysozyme: a model protein for amyloid research. Adv. Protein Chem. Struct. Biol..

[26] Hill S.E., Robinson J., Mathews G., Muschol M. (2009). Amyloid Protofibrils of lysozyme nucleate and grow via oligomer fusion. Biophys. J..

[27] Surmacz-Chwedoruk W., Malka I., Bozycki L., Nieznanska H., Dzwolak W. (2014). On the heat stability of amyloid-based biological activity: insights from thermal degradation of insulin fibrils. PloS ONE.

[28] Cheung A.Y., Al-Atrash J. (1981). Microwave hyperthermia for cancer therapy. Crit. Rev. Bioeng..

[29] Perugia G., Vicini L.P., Collstro F., Gentile V. (2005). Role of hyperthermia in the treatment of peyronie’s disease: a preliminary study. Int. J. Hyperthermia.

[30] Romeyke T., Scheuer H.C., Stummer H. (2015). Fibromyalgia with severe forms of progression in a multidisciplinary therapy setting with emphasis on hyperthermia therapy-a prospective controlled study. Clin Interv Aging.

[31] Almeida J.L.J., Jukemura J., Sampietre S.N., Patzina R.A., Cunha J.E.M., Machado M.C.C. (2006). Effect of hyperthermia on experimental acute pancreatitis. Arq. Gastroenterol..

[32] Huang Y.H., Haegerstrand A., Frostegard J. (1996). Effects of in vitro hyperthermia on proliferative responses and lymphocyte activity. Clin. Exp. Immunol..

[33] Kappel M., Diamant M., Hansen M.B., Klokker M., Pedersen B.K. (1991). Effects of in vitro hyperthermia on the proliferative response of blood mononuclear cell subsets, and detection of interleukins 1 and 6, tumour necrosis factor-alpha and interferon-gamma. Immunology.

[34] Markovic M. (2007). Short term hyperthermia prevents activation of proinflammatory genes in type B synoviocytes by blocking the activation of the transcription factor NF-kB. J. Int. Fed. Clin. Chem..

[35] Stuhlmeier K.M. (2009). Short term hyperthermia prevents the activation of mitogen-activated protein kinase p38. Exp. Gerontol..

[36] Ensor J.E., Crawford E.K., Hansday J.D. (1995). Warming macrophages to febrile range destabilizes tumor necrosis factor-a Mrna without inducing heat shock. Am. J. Physiol..

[37] Wang W., Dow K.E., Flavin M.P. (2008). Hyperthermia amplifies brain cytokine and reactive oxygen species response in a model of perinatal inflammation. Neurosci. Lett..

[38] Walter E.J., Carraretto M. (2016). The neurological and cognitive consequences of hyperthermia. Crit. Care.

[39] Behl C., Schubert D. (1993). Heat shock partially protects rat pheochromocytoma PC12 cells from amyloid B peptide toxicity. Neuosci. Lett..

[40] Johnson G., Refolo L.M., Merril C.R., Wallace W. (1993). Altered expression and phosphorylation of amyloid precursor protein in heat shocked neuronal PC12 cells. Mol. Brain Res..

[41] Tomasovicova N., Hu P.S., Zeng C.L., Majorosova J., Zakutanska K., Kopcansky P. (2019). Dual size-dependent effect of Fe_3_O_4_ magnetic nanoparticles upon interaction with lysozyme amyloid fibrils: disintegration and adsorption. Nanomaterials.

[42] Majorosova J., Schroer M.A., Tomasovicova N., Batkova M., Hu P.S., Kubovcikova M., Svergun D.I., Kopcansky P. (2019). Effect of the concentration of protein and nanoparticles on the structure of biohybrid nanocomposites. Biopolymers.

